# A Case of Cystic Basal Cell Carcinoma Which Shows a Homogenous Blue/Black Area under Dermatoscopy

**DOI:** 10.1155/2011/450472

**Published:** 2010-09-23

**Authors:** Akihiro Yoneta, Kohei Horimoto, Keiko Nakahashi, Satoru Mori, Kazuo Maeda, Toshiharu Yamashita

**Affiliations:** ^1^Department of Dermatology, School of Medicine, Sapporo Medical University, South 1, West 16, Chuo-ku, Sapporo 060-8543, Japan; ^2^Otaru Dermatology Clinic, Otaru 047-0032, Japan

## Abstract

Basal cell carcinoma (BCC) is the most common skin tumor and contains several different histopathological types. Here, we report a case of cystic basal cell carcinoma, which is relatively rare and might be clinically misdiagnosed. A dermatoscopic examination of the case revealed a homogenous blue/black area usually not seen in BCC. We reviewed 102 BCC cases resected and diagnosed at Sapporo Medical University Hospital between April 2005 and March 2010. Among them, only three were the cystic type.

## 1. Case Report

An 80-year-old woman with a nodular lesion on her right breast was referred to our outpatient clinic in January 2010 after she had visited a local dermatology clinic. According to the patient, the lesion had existed since her early childhood, and its size had gradually been increasing. Clinical differential diagnosis included pigmented nevus and adnexal tumors as well as BCC.

**Figure 1:**
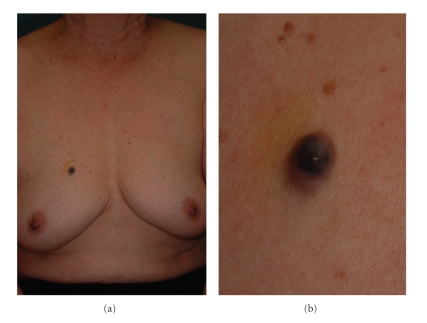
(a) A well-demarcated blue/black nodule was recognized on the right anterior chest. (b) high maginification.

**Figure 2:**
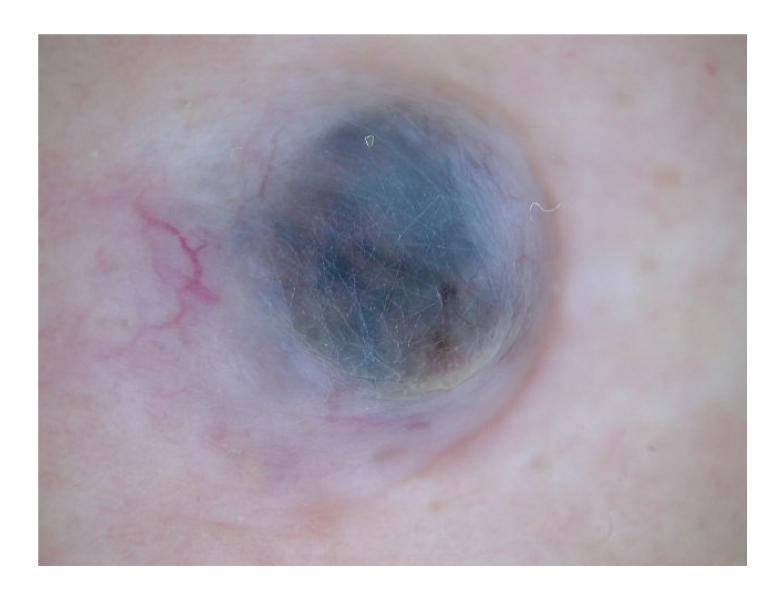
Dermatoscopic examination showed a homogenous blue/black area and arborizing telangiectasia.

A dermatological examination revealed a well-demarcated blue/black colored nodule, measuring 10 × 7.0 cm in size, on her right breast (Figure 1). A dermatoscopic examination showed a homogenous blue/black area in the center of the lesion with arborizing telangiectasia in the periphery to the surrounding region (Figure 2).

**Figure 3:**
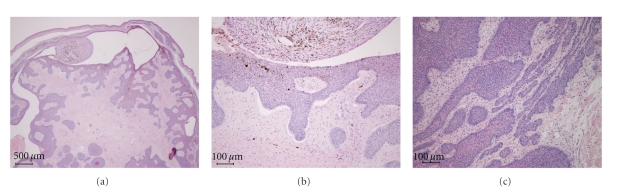
(a) Histological finding showed tumor masses mostly on the dermis with continuation from the epidermis in some parts. The tumor contains cystic spaces. (b) Clefts between the stroma and tumor edge are seen. (c) Palisading of the basaloid cells at the peripheral sites of the tumor masses is noticed.

The tumor was excised with a tumor free margin of 1 cm. Histopathological findings showed tumor masses mostly on the dermis with continuation from the epidermis in some parts. The tumor contained cystic spaces as well as palisading of the basaloid cells at the peripheral sites of the tumor masses and clefts between the stroma and tumor edge, which are often seen in typical basal cell carcinomas (Figure 3).

## 2. Discussion

Basal cell carcinoma is a slowly growing malignant epithelial skin tumor predominantly affecting middle-aged and fair-skinned individuals [1]. Histopathologically, BCCs are composed of islands or nests of basaloid cells, with palisading of the cells at the periphery and a haphazard arrangement of those in the centers of the islands. Various morphological subtypes have been defined. These include solid, micronodular, cystic, multifocal superficial, pigmented, adenoid, infiltrating, sclerosing, keratotic, infundibulocystic, metatypical, basosquamous, and fibroepitheliomatous [2]. Histological differential diagnosis should include trichoblastoma. Criteria that may have value in distinguishing trichoblastomas from BCC include the following: the presence in the former of symmetry, circumscription with smooth margins and “shelling out” of the normal tissue, follicular and “racemiform” patterns of lesional cells, or the lack of a clefting artifact between stroma and epithelium that is characteristic of BCC [3]. In the present case, the tumor masses are relatively asymmetrically distributed, and clefts between the stroma and tumor edge are observed. 

Noninvasive procedures have been developed for the diagnosis of skin cancers [4–7]. Among these, dermatoscopy is the most useful diagnostic procedure with the highest clinical impact in dermatologic practice to better differentiate benign from malignant skin lesions and to detect tumors in the early stage [6, 7]. 

The model for the diagnosis of the pigmented variant of BCC is based on the absence of a pigmented network to differentiate it from melanoma and the presence of at least one positive feature including (1) ulceration (not associated with a recent history of trauma), (2) multiple blue/gray globules, (3) leaf-like areas, (4) large blue/gray ovoid nests, (5) spoke-wheel areas, and (6) arborizing telangiectasia [8]. However, BCC may exhibit a large variety of clinical and dermatoscopic characteristics because of its wide range of histopathological features [9]. 

In the present case, the pigment network was absent and, among the six features, arborizing telangiectasia was present but the other features were not. Instead, a homogenous blue/black area was seen in the center of the tumor, which could be distinguished from the large blue/gray ovoid nests. Histologically, cystic areas overlay the tumor, which may have been the reason for the homogenous blue/black area. The mechanism of the cyst formation was assumed to be massive cell necrosis in the central part of the tumor, which was caused by the rapid tumor growth [1].

**Figure 4:**
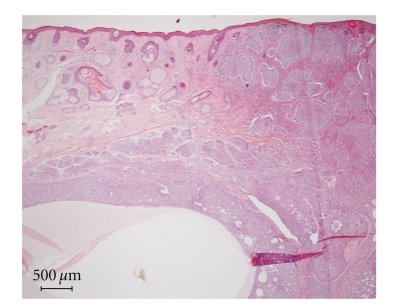
Histological examination showed multiple variably sized nodules with continuation of the epidermis. A cystic area underlies the tumor masses.

**Figure 5:**
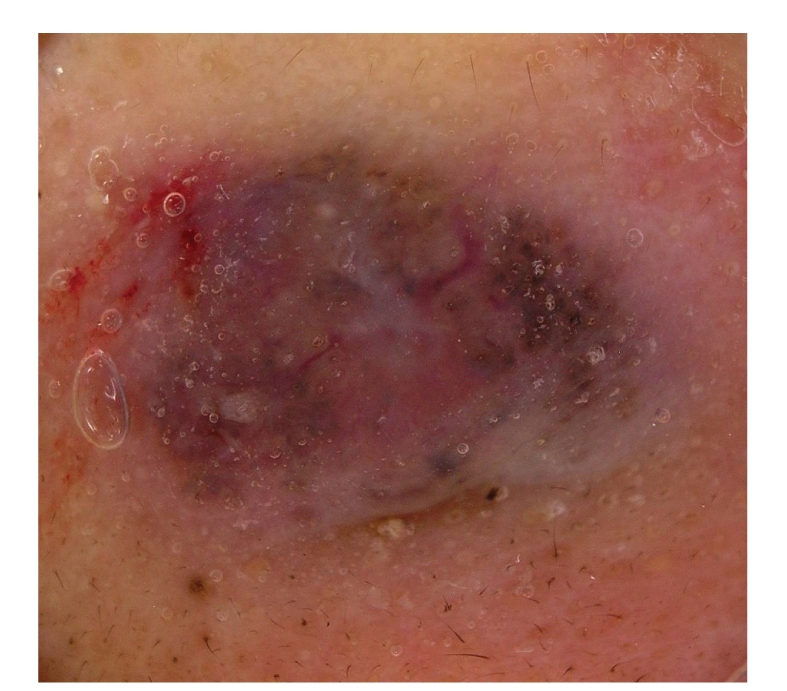
Dermatoscopic examination showed multiple blue/gray globules and arborizing telangiectasia, lacking a homogenous blue/black area.

We evaluated 102 cases of BCC diagnosed at our hospital between April 2005 and March 2010. They were classified into pathological subtypes as shown in Table 1. The solid type was the most frequent in our database as reported elsewhere [2]. The cystic type was relatively rare (2.9%) in the present study (Table 1). Among two other cases of the cystic type, in one, the patient did not undergo a dermatoscopic examination. In the other case, multiple blue/gray globules and arborizing telangiectasia were observed, but there were no homogenous blue/black areas (Figure 4). This may be due to the fact that, unlike the present case, a cystic area existed under tumor masses (Figure 5).

## 3. Conclusions

In conclusion, we herein reported a cystic BCC showing a blue/black nodule on the right chest wall. Dermatoscopy revealed a homogenous blue/black area with arborizing telangiectasia. This rare clinical appearance made it difficult to diagnose; however, our findings suggest that BCC should be considered when a dermatoscopic examination reveals a cystic lesion with a homogenous blue/black area with arborizing telangiectasia.

## Figures and Tables

**Table 1:** Histopathological types of BCC cases at Sapporo Medical University between April 2005 and March 2010.

Histopathological types	Number of cases (%)	
Solid	56	(54.9)
Multifocal superficial	10	(9.8)
Micronodular	8	(7.8)
Sclerosing	8	(7.8)
Adenoid	6	(5.9)
Infiltrating	5	(4.9)
Cystic	3	(2.9)
Fibroepitheliomatous	3	(2.9)
Keratotic	1	(1.0)
Infundibulocystic	1	(1.0)
Basosquamous	1	(1.0)

Total	102	(100)

## Abbreviations

BCC: basal cell carcinoma.
